# Effect of feeding method on nutrient utilization and cow performance in limit-fed cow-calf systems

**DOI:** 10.1093/tas/txab027

**Published:** 2021-02-12

**Authors:** J R Baber, J E Sawyer, L A Trubenbach, T A Wickersham

**Affiliations:** 1 Department of Animal Science, Texas A&M University, College Station, TX 77843, USA; 2 King Ranch^®^ Institute for Ranch Management, Texas A&M University – Kingsville, Kingsville, TX 78363, USA

**Keywords:** beef cattle, feed management, energy restriction, confined feeding

## Abstract

Delivery of limit-fed, complete rations requires significant capital investment, and creates logistical challenges for producers. Deconstruction and separate delivery of roughage and concentrate portions of diets may decrease feeding cost. Two experiments were conducted to evaluate the potential of separately limit-feeding roughage and concentrate. In Experiment 1, 4 ruminally cannulated steers (371 ± 12 kg bodyweight) were used in a 4 × 4 Latin square to evaluate the effects of time of concentrate delivery in deconstructed diets. Intake was restricted to 80% of predicted NE_m_ requirements of a diet consisting of wheat straw (35%), cracked corn (29%), and distillers’ grains (27%) formulated to contain 1.58 Mcal NE_m_/kg. Treatments were: concentrate fed 2 h prior to wheat straw (–2S), concentrate and wheat straw fed as total mixed ration (TMR), concentrate fed 2 h after wheat straw (+2S), and concentrate fed 12 h after wheat straw (+12S). In Experiment 2, 95 mid- to late-gestation cows (503 ± 151 kg) were used in a 112-d trial to evaluate feeding system on cow performance. Cows were assigned to 1 of 12 pens. Treatments were limit-fed the complete diet from Experiment 1 (TMR), fed roughage and concentrate portions of the deconstructed TMR 12 h apart (SEP), and ad libitum bermudagrass hay (HAY). Bodyweight (BW), BCS, and back fat measures were collected every 28 d. In Experiment 1, treatment did not affect DM or OM digestion (*P* ≥ 0.88), rate of particulate passage (*P* ≥ 0.55), or ruminal DM fill (*P* ≥ 0.19). Fill averaged 3.8 kg DM. Nadir of ruminal pH occurred 4–8 h after concentrate was delivered, but mean ruminal pH was not different among treatments (*P* = 0.22) ranging from 6.4 to 6.6 for +2S and 12S, respectively. In Experiment 2, treatment did not affect final BW (518 kg; *P* = 0.72) or final BCS (5.6; *P* = 0.67), but limit-fed strategies tended (*P* = 0.06) to have greater final RE (137.1, 98.9, and –14.6 Mcal for TMR, SEP, and HAY, respectively). Delivering forage and concentrate separately did not change digestion, and timing of concentrate delivery had only minor effects on ruminal fermentation. Limit-feeding a TMR or separate delivery of roughage and concentrate sustained cow performance compared to ad libitum hay consumption.

## INTRODUCTION

Climatic variability in extensive beef production systems is a significant risk, often requiring an increase in inputs (e.g., procurement of expensive feed resources; Tokgoz et al., 2008) or herd depopulation (USDA, 2016) to mitigate its effects. Development of management strategies that enhance production system resiliency improves the sustainability of beef as a global protein source (Darnhofer et al., 2010). One such strategy is the sustainable intensification of beef cow systems, where cows are limit-fed high-energy diets for some portion of the production cycle, or even continuously (Warner et al., 2011; Jenkins et al., 2015). Previous research in our laboratory demonstrated that beef cows limit fed a complete diet (1.54 Mcal NE_m_/kg) at 80% of NE_m_ requirements had reduced apparent maintenance requirements compared to NASEM (2016) predictions (Trubenbach et al., 2019). An additional benefit of limit-fed systems is increased diet digestion (Galyean et al., 1979; Zinn and Owens, 1983; Boardman et al., 2020).

Intensified systems that incorporate limit feeding of a total mixed ration (TMR) potentially bring greater fixed costs to cow-calf production. Coppock (1977) noted that a primary disadvantage of feeding a TMR was the required purchase of equipment (e.g., a grinder to process roughage, a vehicle to mix and deliver feed), such that using a TMR was economically infeasible for smaller producers. These producers are more likely to utilize unprocessed roughages and pre-manufactured concentrate packages to avoid these capital expenses. However, if the limit feeding of separate ingredients alters diet utilization, the cost savings may be obviated. Accordingly, the primary objective of this study was to evaluate a strategy of deconstruction of the TMR into a concentrate package and roughage package as an alternative to a TMR in intensive cow management systems. Specific objectives were to evaluate the effects of these strategies on nutrient utilization, ruminal fermentation, and production outcomes in intensive systems.

## MATERIALS AND METHODS

Experimental protocols were approved by the Institutional Animal Care and Use Committee at Texas A&M University.

### Experiment 1: Diet Utilization

Four steers (371 ± 12 kg of initial BW) fitted with ruminal cannulae participated in a 4 × 4 Latin square design. Steers were housed indoors in individual pens (2.1 × 1.5 m) with *ad libitum* access to water. Diets were limit-fed at 52.88 g/kg BW^0.75^, an amount equivalent to that designed to provide mid-gestation cows with 80% of NASEM (2016) requirements for NE_m_. Diets were constructed from chopped wheat straw, dried distillers’ grains, dry rolled corn, and a mineral supplement (Table 1). Treatments consisted of (1) concentrate package delivered 2 h before hay (–2S), (2) hay and concentrate delivered as a TMR (TMR), (3) concentrate package delivered 2 h after hay (+2S), or (4) concentrate delivered 12 h after hay (+12S). Wheat straw (alone or as a component of TMR) was fed daily at 0730 h.

Each of the four, 20-d experimental periods in the Latin Square comprised 11 d adaptation to treatments (Trubenbach et al., 2019; Boardman et al., 2020), 7 d for intake and digestion measurements, 1 d for ruminal fermentation profile measurement, and 1 d for rumen fill and solid passage measurement. Intake and digestion observations were made on d 12 through 18. Diet samples were collected on d 12 through 17 to correspond with fecal samples collected on d 13 through 18. Fecal bags were placed on steers for a total collection of feces over a 24-h period. Feces within each bag were thoroughly mixed, weighed, and a subsample was collected (5% of fecal matter) and frozen at –20 °C.

Diet, fecal, and ruminal content samples were dried at 55 °C in a forced-air oven for 96 h, allowed to air equilibrate for 24 h, and weighed to determine partial DM. Samples were ground in a Wiley mill (Thomas Scientific, Swedesboro, NJ) to pass a 1 mm screen. Hay and grain samples were composited on an equal air-equilibrated weight basis, while fecal samples were composited by steer across days within the period. Diet, fecal, and ruminal content samples were dried at 105 °C for 24 h to determine DM. Loss of DM following combustion for 8 h at 450 °C was used to determine OM. Analysis of NDF and ADF were performed using an Ankom Fiber Analyzer including amylase but with sodium sulfite omitted and without correction for residual ash (Ankom Technology Corp., Macedon, NY). Direct calorimetry using a Parr 6300 Calorimeter (Parr Instrument Company, Moline, IL) was used to measure gross energy (GE) of feed and fecal samples. Acid detergent insoluble ash (ADIA) was determined by combusting Ankom bags containing ADF for 8 h at 450 °C and weighing the residue.

A suction strainer (Raun and Burroughs, 1962; 19 mm diameter, 1.5 mm mesh) was used to collect ruminal fluid samples prior to feeding (0 h) and at 2, 4, 6, 8, 12, 14, 16, and 20 h after feeding wheat straw. A portable pH meter (Symphony, VWR; Radnor, PA) was used to measure pH immediately upon collection. Before freezing at –20 °C, 8 mL of ruminal fluid was combined with 2 mL of 25% *m*-phosphoric acid containing 2-ethyl butyrate as an internal standard for subsequent VFA analysis. On d 20 of each period, ruminal contents were completely removed by manual evacuation prior to (0 h), and at 4 and 12 h after feeding wheat straw. Extrusa were weighed, three subsamples (500 g) were collected, and the remaining material was returned to the rumen. Ruminal fluid samples were thawed and centrifuged at 20,000 × *g* for 10 min at room temperature. Concentrations of VFA were measured using a gas chromatograph (Hewlett-Packard Mod. 5890; Avondale, PA) according to Vanzant and Cochran (1994).

### Experiment 2: Cow Performance

Ninety-five dry, mid- to late-gestation crossbred cows (503 ± 151 kg) were used in an experiment to compare alternative feed delivery systems to conventionally hay fed. Treatments were (1) limit feeding a TMR (TMR); (2) feeding the concentrate and wheat straw components of the TMR separately but in the same proportions as in the TMR (SEP), equivalent to 12S from Experiment 1; and (3) ad libitum access to bermudagrass hay (HAY; Table 1). The TMR and SEP diets were the same as those fed in Experiment 1. Both TMR and SEP were fed (g/kg EBW^0.75^) to provide 80% of predicted NE_m_ requirements according to NASEM (2016; Table 2). Cows fed HAY were allowed ad libitum consumption, rather than being limit-fed.

**Table 1.** Ingredient and nutrient composition of diets used in Experiments 1 and 2

Ingredient, % as fed	Limit-fed diet^*a*^	HAY^*b*^
Bermudagrass hay		100.00
Wheat Straw	34.52	
Corn	29.46	
Distillers’ grains	27.46	
Mineral	2.46	
Urea	1.10	
Molasses	5.00	
Nutrient composition, % DM basis		
OM	92.80	91.31
CP	16.50	7.72
NDF	48.17	74.20
ADF	26.20	41.40
ME, Mcal/kg^*c*^	2.47	2.04
NE_m_, Mcal/kg^*c*^	1.56	1.19

^*a*^ Limit-fed diet fed to all treatments in Experiment 1 and limit-fed systems in Experiment 2.

^*b*^ Bermudagrass hay was fed ad libitum to conventional hay system in Experiment 2.

^*c*^ Estimated using NASEM (2016).

**Table 2.** Intake, energy intake, and feed costs per cow of each cow-calf production system

Item	Treatment^*^		
	HAY	SEP	TMR
Feed offered, kg AF/d	14.85	5.58	5.58
Feed cost, $/kg	0.14	0.17	0.17
Total feed cost, $/d	2.10	0.99	0.99
Feed waste, kg AF/d	1.48		
Estimated DMI	11.83	4.97	4.97
ME consumed, Mcal/d	24.13^a^	12.62^b^	12.62^b^
Feed cost, $/Mcal consumed	0.09	0.08	0.08

^*^HAY = cows fed ad libitum hay; SEP = cows limit fed concentrate 12 h after wheat straw; TMR = cows limit fed total mixed ration once daily.

^a^ Calculated from estimated DMI and NASEM (2016) ME concentration for bermudagrass hay.

^b^ Calculated from estimated DMI and ME concentration found in experiment 1.

Cows were stratified by body weight, week of gestation, body condition score, and age, and within strata were randomly assigned to a pen. Pens (*n* = 4 per treatment) were randomly assigned to treatments and served as the experimental unit. Cows were fed from approximately d 120 to 240 of gestation and had *ad libitum* access to water. Prior to the start of the feeding period (d –6), cows were weighed to establish amounts to be fed and treatment application was initiated to allow for equilibration of gut fill. Measurements of body weight and body condition score (BCS; the scale of 1 to 9; 1, emaciated; 9, obese; an average of three trained personnel) were collected prior to feeding on d 0, 28, 56, 84, and 112. Body condition scores were used for both direct comparisons and as a component of estimation of body energy reserves.

After d 112, cows returned to a common pasture to calve and were managed as a single group. Calving started approximately 45 d after turnout, and calf birth weight and cow BCS at calving were recorded. Additionally, calf BW, cow BW, and cow BCS were collected approximately 45 d after birth.

### Calculations

#### Experiment 1: Digestion.

Digestion coefficients were calculated using:


$$\begin{array}{l} \left[1\;\;\left(nutrient\;output/nutrient\;intake\right)\;\times \;100\right] \end{array}$$

Average DM fill was calculated using the following equation:


$$\begin{array}{l} DM\;fill=\frac{DM\;Fill_{0}+DM\;Fill_{4}+DM\;Fill_{12}}{3} \end{array}$$

where: *DM Fill* = Average DM fill, kg

*DM Fill*_*0*_ = Rumen evacuation dry matter contents before wheat straw fed

*DM Fill*_*4*_ = Rumen evacuation dry matter contents 4 h after wheat straw fed

*DM Fill*_*12*_ = Rumen evacuation dry matter contents 12 h after wheat straw fed

Passage rate per hour (Waldo et al., 1972) was calculated using ADIA and the following equation:


$$\begin{array}{l} PR\;=\;\frac{\left[\frac{ADIAin}{ADIArumen}\right]}{24} \end{array}$$

where *PR* = solid passage rate, %/h

*ADIA*_*in*_ = intake of ADIA, kg

*ADIA*_*rumen*_ = average of ADIA (kg) amount at rumen evacuation at h 0, 4, and 12

#### Experiment 2: cow performance.

Empty body energy was calculated using equations published in Nutrient Requirement of Beef Cattle (NASEM 2016). Body composition was estimated using the following equations:


$$\begin{array}{l} AF\;=\;3.768\;\times \;CS \end{array}$$

$$\begin{array}{l} AP\;=\;20.09\;0.668\;\times \;CS \end{array}$$

where: *AF* = proportion of empty body fat, %

*AP* = proportion of empty body protein, %

*CS* = body condition score


$$\begin{array}{l} TF\;=\;AF\;\times \;EBW \end{array}$$

$$\begin{array}{l} TP\;=\;AP\;\times \;EBW \end{array}$$

$$\begin{array}{l} SBW\;=\;0.96\;\times \;BW \end{array}$$

$$\begin{array}{l} EBW=\;0.891\;\times \;SBW \end{array}$$

where: *TF* = total fat, kg

*TP* = total protein, kg

*BW* = body weight, kg

*SBW* = shrunk weight, kg

*EBW* = empty body weight, kg


$$\begin{array}{l} TBE\;=\;9.4\;\times \;TF\;+\;5.7\;\times \;TP \end{array}$$

where: *TBE* = total body energy, Mcal


$$\begin{array}{l} RE\;=\;TBEf\;-\;TBEi \end{array}$$

where

*TBE*_*f*_ = total body energy at end of the period, Mcal

*TBE*_*i*_ = total body energy on d 0, Mcal

*RE* = retained energy, Mcal

### Statistical Analysis

#### Experiment 1: digestion.

Intake, digestion and ruminal passage parameters were analyzed using the MIXED procedure of SAS 9.3 (SAS Inst. Inc., Cary, NC). Terms in the model included treatment and period with a steer as a random effect. Volatile fatty acid and pH responses were analyzed as repeated measures using the MIXED procedure; model effects included treatment, hour, and hour × treatment. The repeated term was sampling hour, the subject effect was treatment × steer, and the specified covariance structure was compound symmetry, selected based on the Bayesian Information Criterion. Least squares means were estimated and separated using t-tests when protected by a significant *F*-test of the effect. For all tests, significance was determined at alpha ≤0.05.

#### Experiment 2: cow performance.

Measures of cow BW, BW changes, BCS, BCS changes, backfat thickness, and RE were analyzed using MIXED procedure of SAS 9.3 (SAS Inst., Cary, NC). The fixed effect term in the model was treatment and the random effect (error term) was pen within the treatment. Standard deviation of BW, BW change, and BCS within a pen was analyzed using the MIXED procedure of SAS 9.3 (SAS Inst., Cary, NC). Terms in the model included treatment and the random effect (error term) pen within the treatment. Means were estimated and compared as in Experiment 1.

## RESULTS

### Experiment 1: Digestion and Ruminal Fermentation

Nutrient intakes were similar among all treatments, as designed (Table 3; *P* ≥ 0.52), and no differences in the digestion of DM, OM, NDF, ADF, or GE were observed (*P* ≥ 0.73). Molar concentration of total VFA did not differ among treatments (*P* = 0.65) and averaged 81.10 mM.

**Table 3.** Effect of time concentrate is offered on nutrient intake and digestibility in steers consuming wheat straw and concentrate separately or as a component of a TMR (Experiment 1)

	Treatment^*a*^					*P*-value^*b*^
Item	-2S	TMR	+2S	+12S	SEM	
No. of observations	4	4	4	4		
Intake, kg/d						
DM	3.34	3.34	3.34	3.34	0.04	0.67
OM	3.10	3.10	3.10	3.10	0.03	0.67
NDF	1.62	1.62	1.62	1.62	0.02	0.52
ADF	1.00	1.00	1.00	1.00	0.01	0.96
GE, Mcal/d	14.84	14.84	14.84	14.84	0.16	0.83
DE, Mcal/d	10.31	10.29	10.40	10.44	0.18	0.89
Total tract digestion, %						
DM	68.35	68.32	68.81	69.27	1.16	0.99
OM	71.07	71.17	71.74	72.09	1.12	0.88
NDF	61.08	62.70	62.55	62.07	1.68	0.90
ADF	51.01	52.88	53.89	53.68	2.12	0.73
GE	69.46	69.38	70.04	70.31	1.08	0.90

^*a*^–2S = concentrate fed 2 h before wheat straw; TMR = concentrate and wheat straw fed as TMR; +2S = concentrate fed 2 h after wheat straw; +12S = concentrate fed 12 h after wheat straw.

^*b*^ Treatments with different superscripts differ (*P* < 0.05).

**Figure 1.**
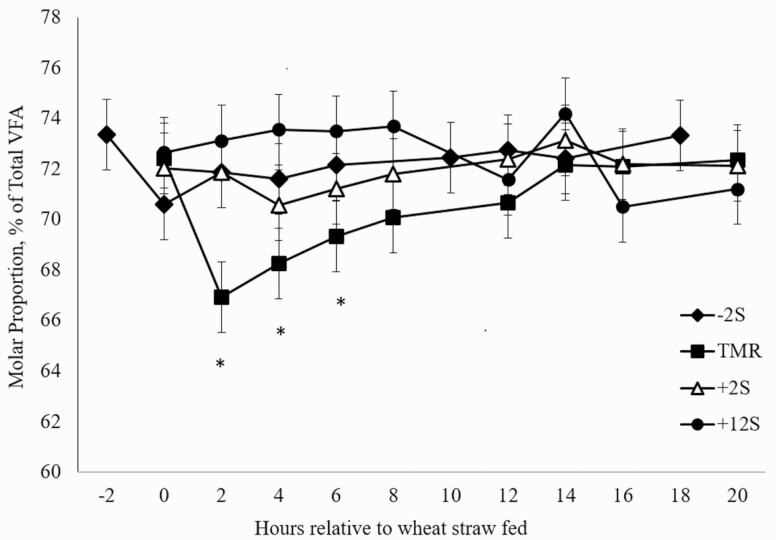
Effect of time of concentrate delivery on acetate production over time in steers consuming wheat straw. –2S = concentrate fed 2 h before wheat straw; TMR = concentrate and wheat straw fed as TMR; +2S = concentrate fed 2 h after wheat straw; +12S = concentrate fed 12 h after wheat straw. Significant effects of time (*P* = 0.04) and treatment × time (*P* = 0.01). * denotes time points where treatments differ.

**Figure 2.**
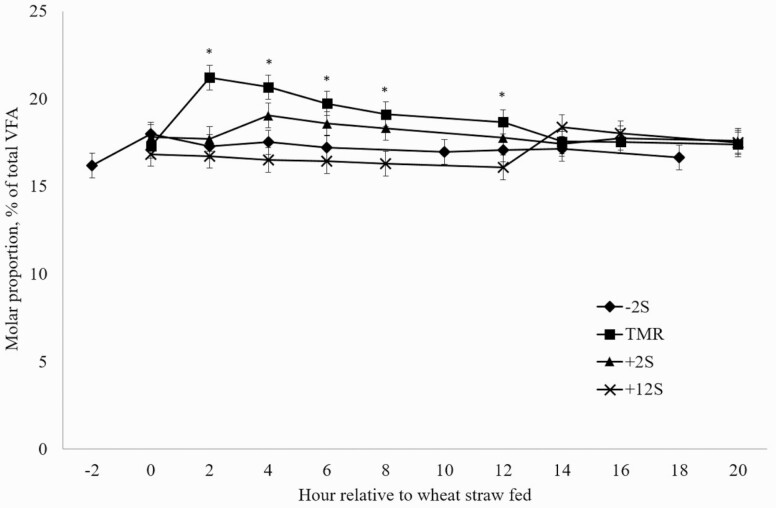
Effect of time of concentrate delivery on propionate production over time in steers consuming wheat straw. –2S = concentrate fed 2 h before wheat straw; TMR = concentrate and wheat straw fed as TMR; +2S = concentrate fed 2 h after wheat straw; +12S = concentrate fed 12 h after wheat straw. Significant effects of time (*P* = 0.08) and treatment × time (*P* = 0.04). * denotes time points where treatments differ.

**Figure 3.**
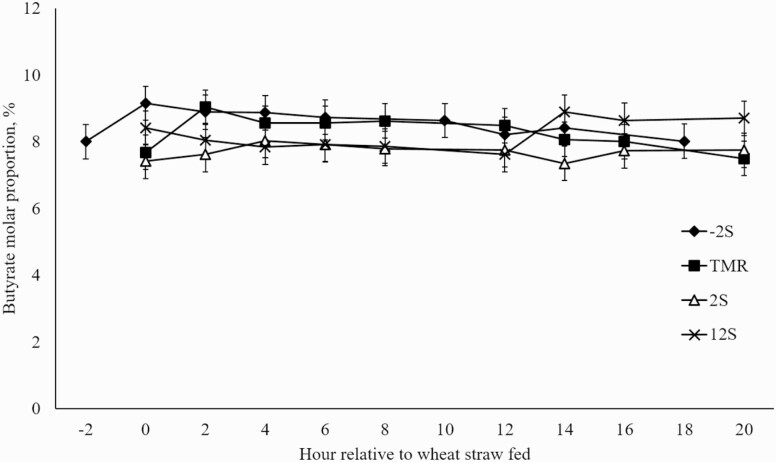
Effect of time of concentrate delivery on butyrate production over time in steers consuming wheat straw. –2S = concentrate fed 2 h before wheat straw; TMR = concentrate and wheat straw fed as TMR; +2S = concentrate fed 2 h after wheat straw; +12S = concentrate fed 12 h after wheat straw. Significant effects of treatment × time (*P* = 0.01).

**Figure 4.**
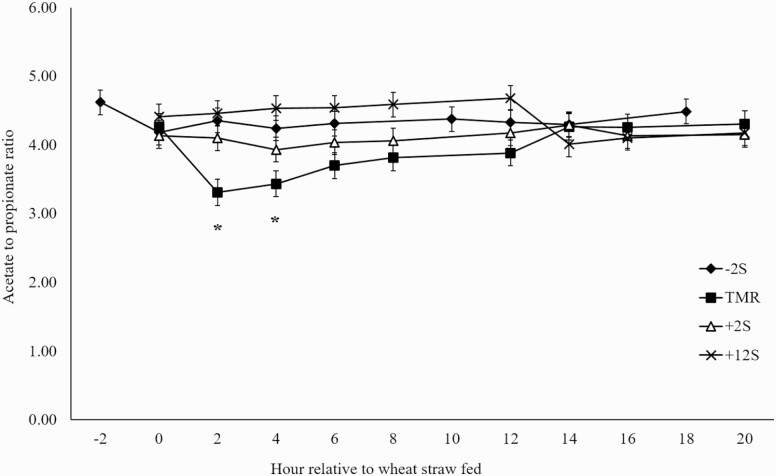
Effect of time of concentrate delivery on acetate to propionate ratio over time in steers consuming wheat straw. –2S = concentrate fed 2 h before wheat straw; TMR = concentrate and wheat straw fed as TMR; +2S = concentrate fed 2 h after wheat straw; +12S = concentrate fed 12 h after wheat straw. Significant effects of time (*P* = 0.31) and treatment × time (*P* = 0.08). * denotes time points where treatments differ.

Differences in mean ruminal acetate proportions between treatments were not observed (*P* = 0.53; Figure 1). Significant effects of time (*P* = 0.04) and treatment × time interaction (*P* = 0.01) were observed for ruminal acetate proportions, driven by reductions in acetate proportions occurring after feeding concentrate for each treatment. Additionally, there was a significant treatment × time interaction (*P* = 0.04) and a tendency for an effect of time (*P* = 0.08) in propionate proportions (Figure 2). Conversely to acetate proportions, an increase in propionate proportions occurred after concentrate was fed. A treatment × time interaction (*P* = 0.01) was observed for butyrate proportions (Figure 3) with proportions increasing slightly 2 h after concentrate delivery. Molar proportions of butyrate, isobutyrate, isovalerate, and valerate were not affected by treatment (P > 0.26) and averaged 8.19, 0.77, 0.88, and 0.59, respectively. Acetate:propionate ratios were not different among treatments (*P* = 0.23) and were 4.35, 3.91, 4.11, and 4.38 for –2S, TMR, +2S, and +12S, respectively. There was a tendency for a treatment × time interaction (*P* = 0.08) for acetate:propionate ratio (Figure 4), resulting from the changes in each VFA relative to the timing of concentrate feeding.

**Figure 5.**
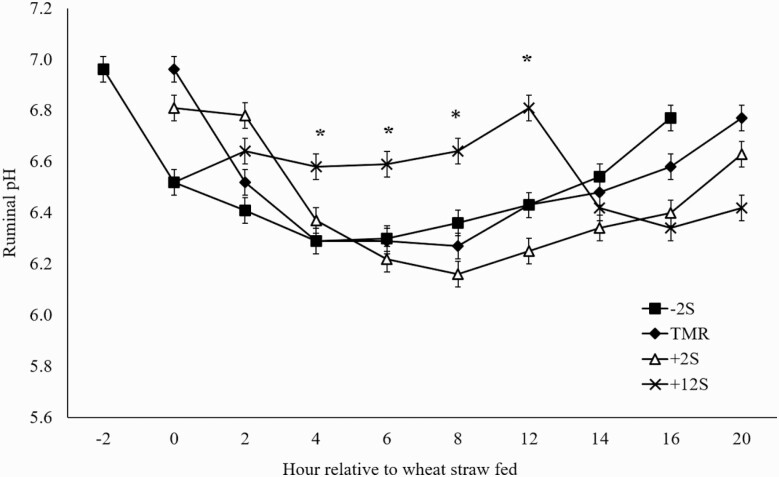
Effect of time of concentrate delivery on ruminal pH over time in steers consuming wheat straw. –2S = concentrate fed 2 h before wheat straw; TMR = concentrate and wheat straw fed as TMR; +2S = concentrate fed 2 h after wheat straw; +12S = concentrate fed 12 h after wheat straw. Significant effects of time (*P* < 0.01) and treatment × time (*P* < 0.01). * denotes time points where treatments differ.

There were significant time and treatment × time interaction effects on ruminal pH (Figure 5; *P <* 0.01). A pH nadir was observed approximately 4–8 h after concentrate was fed for each treatment, but across time treatment means for ruminal pH were not different (*P* = 0.22). Ruminal fill of DM or ADF was not affected by treatment (*P* > 0.19; Table 4). Passage rates were similar between treatments (*P* = 0.55).

**Table 4.** Effect of time concentrate is offered on rumen fill and passage in steers consuming wheat straw and concentrate separately or as a component of a TMR (Experiment 1)

	Treatment^*a*^					
Item	–2S	TMR	+2S	+12S	SEM	*P*-value
No. of observations	4	4	4	4		
Fill, kg/d						
DM	3.45	3.71	3.78	4.10	0.27	0.24
ADF	1.49	1.58	1.64	1.77	0.14	0.19
Passage Rate, %/h	2.33	2.23	2.31	2.06	0.14	0.55

^*a*^ –2S = concentrate fed 2 h before wheat straw; TMR = concentrate and wheat straw fed as TMR; +2S = concentrate fed 2 h after wheat straw; +12S = concentrate fed 12 h after wheat straw.

### Experiment 2: Cow Performance

Feeding system did not affect initial (d 0) or final BW (d 112) (Table 5; *P* ≥ 0.22). Weight change in cows fed HAY from d 0 to 28 was greater than in those fed using SEP or TMR systems (*P* < 0.01), and weight loss in cows fed SEP tended to be different from those fed TMR (*P* = 0.08). In the first 28 d, cows fed HAY (positive) and SEP (negative) had weight changes that were different from zero (*P* ≤ 0.03); those fed TMR (negative) tended to differ from 0 (*P* = 0.08). Weight change from d 29 to 56 was not different between treatments (*P* = 0.96), but period weight changes were positive (different from zero; *P* < 0.01) for all treatments. From d 57 to 84, cows fed HAY lost weight compared to those fed SEP or TMR (*P* ≤ 0.03). In the final 28-d period, cow fed SEP and TMR gained more BW than those fed HAY (*P* < 0.01), for whom gain was not different than zero (*P* = 0.72).

**Table 5.** Effect of feeding method on cumulative and period body weight and body condition score (BCS)^*a*^ in mid- to late-gestation cows

Item	Treatment^*b*^			SEM	*P*-value^*c*^
	HAY	SEP	TMR		
No. of pens	4	4	4		
Initial BW (d 0), kg	518.6	496.6	492.4	10.72	0.22
Period weight changes, kg					
d 0–28	6.8^a*^	–12.4^b*^	–5.1^b^	2.66	<0.01
d 29–56	7.6^*^	7.4^*^	6.8^*^	2.12	0.96
d 57–84	–7.3^a^	5.6^b^	7.8^b^	3.59	0.03
d 85–112	1.0^a^	16.0^b*^	13.5^b*^	3.82	<0.01
Change from initial body weight, kg					
d 28	6.8^a*^	–12.4^b*^	–5.1^b^	2.66	<0.01
d 56	14.4^a*^	–4.9^b^	1.7^b^	3.33	<0.01
d 84	7.1	0.6	9.5	4.36	0.37
d 112	8.1	16.6^*^	22.9^*^	5.15	0.17
Final BW (d 112), kg	526.7	513.1	515.3	12.69	0.72
Initial BCS (d 0)	5.71	5.45	5.47	0.09	0.14
Period BCS changes					
d 0–28	–0.04	–0.13	–0.07	0.08	0.73
d 29–56	–0.01	0.15^*^	0.07	0.06	0.21
d 57–84	–0.14^*^	–0.01	0.02	0.05	0.07
d 85–112	–0.06	0.11	0.07	0.07	0.24
Change from initial BCS					
d 28	–0.04	–0.13	–0.07	0.08	0.73
d 56	–0.05	0.02	–0.01	0.09	0.88
d 84	–0.19^*^	0.01	0.02	0.08	0.19
d 112	–0.25^a*^	0.12^b^	0.09^b^	0.07	0.01
Final BCS (d 112)	5.46	5.58	5.56	0.09	0.67

^*a*^Body condition score: 1 = emaciated; 9 = obese.

^*b*^HAY = cows fed ad libitum hay; SEP = cows limit fed concentrate 12 h after wheat straw; TMR = cows limit fed total mixed ration once daily.

^*c*^Treatments with differing superscripts differ (*P* < 0.05).

*Treatment is significantly different from zero (*P* < 0.05).

Despite variation in period weight gains, cumulative weight changes from d 0 to 112 did not differ (*P* = 0.17) among treatments, and were 8.1, 16.6, and 22.9 kg for cows fed HAY, SEP, and TMR, respectively. Total weight change in cows fed SEP and TMR were positive (*P* ≤ 0.01), but weight change in those fed HAY did not differ from zero (*P* = 0.15).

Initial BCS (*P* = 0.14) and final BCS (*P* = 0.67) were similar among treatments (Table 5), and within-period changes in BCS were not separable among treatments for any time interval (*P* ≥ 0.07). However, cumulative change in BCS (d 0–112) for cows fed HAY (–0.25) was negative (*P* = 0.01) and different (*P* < 0.01) from BCS change in cows fed SEP (0.12) and TMR (0.09), which were not different from zero change (*P* > 0.13).

Retained energy was estimated using BCS and BW (Table 6). During the first 28 d of the trial, cows fed HAY retained more energy than those fed SEP and TMR (*P* ≤ 0.05). Energy retention increased in cows on all treatments on d 56 and 84, and was similar among treatments (*P* > 0.26), with none different from zero (*P* ≥ 0.08) suggesting that cows were at maintenance. By d 112, there was a tendency (*P* = 0.06) for a treatment effect on RE; in cows fed HAY, retained energy was not different from zero (*P* = 0.72), while cows fed SEP and TMR had positive values of RE (greater than zero, *P* ≤ 0.04).

**Table 6.** Effect of feeding method on total retained energy estimated from body condition score (BCS)^*a*^ in mid- to late-gestation cows

Item	Treatment^*b*^			SEM	*P*-value^*c*^
	HAY	SEP	TMR		
No. of pens	4	4	4		
RE from BCS, Mcal					
d 28	28.58^a^	–103.53^b*^	–46.76^b^	23.97	0.01
d 56	65.54	–17.77	13.01	33.79	0.26
d 84	24.79	11.16	56.36	34.97	0.65
d 112	–14.61	98.86^*^	137.05^*^	40.01	0.06

^*a*^ Body condition score: 1 = emaciated; 9 = obese.

^*b*^ HAY = cows fed ad libitum hay; SEP = cows limit-fed concentrate 12 h after wheat straw; TMR = cows limit-fed total mixed ration once daily.

^*c*^ Treatments with differing superscripts differ (*P* < 0.05).

Within pen standard deviation is presented as a method of evaluating variation that may have been affected by treatments for BW and BCS (Table 7) due to intrapen variation in feed intake as a consequence of limited feed allocation in a group environment. There was a tendency (*P* = 0.08) for lower standard deviation in initial BW (d 0) for pens fed SEP than those fed TMR and HAY. On d 28, this tendency disappeared and no differences in the intrapen standard deviation of BW between treatments were observed (*P* = 0.28). However, by d 56, intrapen standard deviation of BW was greater for pens fed SEP and TMR than those fed HAY (*P* ≤ 0.04). Similarly, pens receiving TMR had a greater (*P* ≤ 0.01) intrapen standard deviation in BW on d 84 than those receiving HAY and SEP. Standard deviation of final BW (d 112) was greater for pens receiving TMR than for those receiving HAY (62.3 kg; *P* = 0.02), but pens fed SEP had standard deviation of BW intermediate to and not different from HAY or TMR (*P* ≥ 0.13).

**Table 7.** Effect of feeding method on within-pen standard deviation of performance characteristics in mid- to late-gestation cows

Item	Treatment^*b*^			SEM	*P*-value^*c*^
	HAY	SEP	TMR		
No. of pens	4	4	4		
Standard deviation of body weight					
d 0	55.6	65.3	66.2	3.20	0.08
d 28	61.3	66.9	68.9	3.23	0.28
d 56	60.7^a^	69.7^b^	75.3^b^	2.58	0.01
d 84	62.9^a^	62.5^a^	79.3^b^	3.50	0.01
d 112	62.3^a^	71.4^ab^	83.3^b^	5.05	0.05
Standard deviation of change from initial body weight					
d 28	13.2	11.6	12.9	1.16	0.61
d 56	15.5	19.0	19.5	2.78	0.57
d 84	16.0^a^	23.5^ab^	26.1^b^	2.58	0.05
d 112	18.8	29.6	31.3	3.55	0.07
Standard deviation of BCS^*b*^					
d 0	0.52	0.48	0.54	0.08	0.87
d 112	0.47	0.50	0.57	0.08	0.72
Change	-0.05	0.02	0.02	0.06	0.62

^*a*^ HAY = cows fed ad libitum hay; SEP = cows limit-fed concentrate 12 h after wheat straw; TMR = cows limit-fed total mixed ration once daily.

^*b*^ Body condition score: 1 = emaciated; 9 = obese.

^*c*^ Treatments with differing superscripts differ (*P* < 0.05).

*Treatment is significantly different from zero (*P* < 0.05)

Within pen standard deviation was also calculated for the change from initial BW (Table 7). On d 28 and 56, the intrapen standard deviation of BW change from initial BW was not different among treatments (*P* > 0.57), but on d 84 was greater for pens fed TMR compared to those fed HAY (*P* = 0.02). On d 84, intrapen standard deviation of weight change in pens fed SEP was intermediate to and not different from the others (*P* > 0.15). There was a tendency (*P* = 0.07) on d 112 for intrapen standard deviation of BW change to be greatest for pens fed TMR and least for pens fed HAY. Standard deviation of BCS on d 0 and 112 did not differ between treatments (*P* > 0.72), nor did the standard deviations of BCS change from d 0 to 112 (*P* = 0.62).

Cows started calving approximately 45 d after the feeding trial ended, and average cow BCS at calving and calf birth weights are reported in Table 8. No differences were observed between treatments for cow BCS at calving (5.0 on average; *P* = 1.00) or calf birth weight (33.7 kg on average; *P* = 0.36). Cow BW, cow BCS, and calf BW 45 d after calving, were not different among treatments (*P* > 0.66) and averaged 486.4 kg, 4.8, and 74.2 kg, respectively.

**Table 8.** Effect of feeding method on cow body weight, cow body condition score (BCS)^*a*^, and calf performance after limit-feeding period

Item	Treatment^*b*^			SEM	*P*-value^*c*^
	HAY	SEP	TMR		
No. of pens	4	4	4		
At calving					
Cow BCS	5.0	5.0	5.0	0.13	1.00
Calf birth weight, kg	32.7	35.0	33.5	1.05	0.36
45 d after calving					
Cow body weight, kg	481.9	491.5	486.0	11.27	0.83
Cow BCS	4.7	4.8	4.8	0.11	0.74
Calf body weight, kg	71.8	76.5	74.2	3.56	0.66

^*a*^ Body condition score: 1 = emaciated; 9 = obese.

^*b*^ HAY = cows fed ad libitum hay; SEP = cows limit-fed concentrate 12 h after wheat straw; TMR = cows limit-fed total mixed ration once daily.

^*c*^Treatments with differing superscripts differ.

## DISCUSSION

To facilitate the implementation of limit-fed production systems by any sizes of a cow-calf operation, experiment 1 was designed to evaluate the effects of alternative feeding methods on nutrient utilization and fermentation using limit-fed steers as a model. Treatments were designed to reflect options available to a producer without the capabilities to feed a TMR. In those situations, a producer would feed one diet component (hay or concentrate) and then the other (–2S or +2S), or feed one component in the morning and the other in evening (+12S).

No differences in the digestion of DM, OM, NDF, or ADF were found in our study. In cows fed ad libitum, DM digestion of a complete diet was reduced compared to cows fed the same ingredients separately (Gordon et al., 1995). Improved DM and OM digestibility were also observed in cows fed separate ingredients compared to a TMR when intake was limited to 2.5% BW (Phipps et al., 1984), although that degree of restriction is likely close to ad libitum feeding. The relatively higher feeding rates in these studies compared to the present study allowed some selectivity among separately fed ingredients, which resulted in improved digestibility when cattle were allowed separate access to ingredients. Cattle do not selectively consume feed when fed at more restrictive levels of intake (i.e., all feed offered is consumed). In our study, where all feed offered was consumed, there was no opportunity for changes in diet composition to affect measures of digestion.

Differences were not observed between treatments for GE digestibility, and DE and ME were calculated from digestibility of GE. When this diet was fed at the same level of restriction, Trubenbach et al. (2019) and Boardman et al. (2020) observed OM digestion (71.7% and 76.7%, respectively) and GE (68.6% and 75.2%, respectively) compared to those in the present study. In our experiment, energy availability was not altered by the time concentrate was delivered (2.54 Mcal ME/kg DM), but was approximately 10% greater than observed in the prior studies (Trubenbach et al., 2019; Boardman et al., 2020; 2.18 and 2.38 Mcal ME/kg DM, respectively). Tabular values from NASEM (2016) suggest ME availability to be 2.45 Mcal/kg DM for this diet, which is lower than the observed ME availability in this experiment. As intake departs further from ad libitum consumption, digestibility and energy availability per unit of diet have a greater deviation from predicted values (Murphy and Loerch, 1994).

Feeding large quantities of concentrates can cause acidosis and other metabolic issues (Tremere et al., 1968; Krause and Oetzel, 2006), and ruminal pH is often used as an indicator of risk for acidosis. The pH changes in this study were relative to the timing of feeding the concentrate portion of the diet, as indicated by the treatment by time interaction. However, it is important to note that ruminal pH was never less than 6 for any treatment or time point, suggesting acidosis was not a concern feeding these diets at restricted intake levels, even when concentrate and roughage components were fed separately. Compared to all other treatments +12S had reduced pH variation throughout the day, with lower peak and greater nadir of ruminal pH. Offering more meals throughout the day reduces ruminal pH fluctuations (Kaufmann, 1976), and in the present study, the combination of increased time between meals for the +12S and the relatively limited amount of substrate provided likely mitigated pH extremes. Overall, mean pH was not different when comparing treatments, similar to Yan et al. (1998), who observed no differences in ruminal pH when cattle were fed a ration separated into forage and concentrate components or consuming a total mixed ration.

Although molar concentrations of total VFA were not affected by treatment, the proportion of total VFA for acetate numerically decreased 2 h after delivery of the concentrate component of the diet. This resulted in the treatment × time interaction observed for acetate proportions in experiment 1. However, when the concentrate was fed for +12S, acetate proportion was numerically increased. Proportions of propionate for all treatments numerically increased when the concentrate was delivered 2 h after concentrate was delivered. Similar to our results, Phipps et al. (1984) reported no differences in proportions of acetate, propionate or butyrate when ingredients were combined or separated at restricted intake levels for dairy cattle. Also in agreement with our results, Yan et al. (1998) found no differences in proportions of VFA or total VFA concentration when cows were fed a complete diet or separately fed the ingredients of that diet. Treatment × time interaction for acetate:propionate ratios were driven by time of concentrate delivery as well. Mean acetate:propionate ratio (4.18) was greater in our experiment than those observed by Trubenbach et al. (2019) and Boardman et al. (2020), 2.38 and 3.07, respectively. Ruminal pH results in our study suggest a more conducive environment for the rumen microbial population to ferment and digest substrate compared to previous studies.

Changes in digestion due to limiting intake may be in part due to changes in passage rate. In this study, intake was similar among treatments, and no differences in passage rates, DM fill or ADF fill were observed. Reducing intake of a similar diet from 120% to 80% of predicted maintenance requirements reduced particulate passage rate from 2.44% to 1.88 %/h (Boardman et al., 2020); the rate observed in the current study was more similar to that of the higher intake level in that trial, which may be due to adding a feeding bout rather than the total volume of intake.

Experiment 1 found no detrimental effects on digestion or ruminal fermentation from separating a TMR into its concentrate and roughage components. Experiment 2 was designed to compare the performance of cows using these alternative feeding methods for limit-fed cow-calf production systems to a more conventional cow-calf system feeding hay. Separated concentrate and roughage components were fed 12 h apart for SEP because this treatment (+12S) tended to be most distinct from TMR in experiment 1.

To remove the effects of fill adjustment during the experimental period, treatments were applied 6 d prior to starting experiment 2. Although initial (d 0) BW were statistically similar among treatments, limit-fed treatments were numerically lower than HAY by approximately 24 kg, presumably due to filling differences. Some bodyweight losses in cows on limit-fed treatments were expected in the first 28 d (Boardman et al., 2020), as cows adjusted to a new maintenance equilibrium (Freetly et al., 2006). Cows fed HAY gained a small amount of BW (1.3% of initial BW) while those being limit fed lost 1.7% of initial BW during this 28-d period; these changes were accompanied by insignificant losses in body condition for all treatments. However, over the course of the 112-d feeding period, cows on limit-feeding programs gained BW relative to initial weight and had positive RE, suggesting that the anticipated adaptation to the limit feeding systems occurred regardless of how diets were provided.

Bodyweight and retained energy measures were not different from zero for cows fed hay ad libitum, suggesting that they were at maintenance, while those being limit-fed had positive RE at d 112. These results are consistent with the adaptions observed to diet restriction in beef cows (Freetly et al., 2006), including that limit-fed corn or fed hay ad libitum (Loerch, 1996) or those fed diets similar to those used in the current study at 80% of predicted maintenance (Trubenbach et al., 2019, Boardman et al., 2020). Limit feeding, either as a TMR or feeding a limited amount of hay followed by a concentrated package, resulted in more efficient diet utilization for cow maintenance; those being limit-fed gained weight and retained energy regardless of feeding method.

Retained energy was estimated according to NASEM (2016) equations for body energy content based on BCS and BW, which resulted in treatment effects on RE corresponding to effects on BW and BCS change. Following treatment application, a period of adjustment may have occurred resulting in differences in apparent RE among the treatments applied, especially between cows fed HAY and those on limit-fed treatments (SEP or TMR). Such adjustments have been reported previously (Freetly and Nienaber, 1998; Trubenbach et al., 2019) and may occur rapidly following a diet restriction (Freetly et al., 2006).

In cows receiving limit-fed treatments, RE accumulated as the trial progressed, while in cows fed HAY *ad libitum* it declined, so that by the end of the 112-d period cows fed HAY appear to have been at maintenance (RE was not different from zero) while those receiving either limit-fed treatment had positive RE. This outcome is important, as it suggests that feeding a complete (TMR) or deconstructed diet (SEP) resulted in similar efficiencies of diet utilization, which appeared to be greater than that in cows fed HAY. This is especially apparent in the context of the total amount of ME delivered per day (see Table 2); cows fed HAY received nearly twice the daily amount of ME compared to cows fed TMR or SEP. While it is possible that ME value of HAY was overestimated, and/or that hay waste was not fully accounted for, it still remains that using either a complete diet or a deconstructed diet in a limit-fed system resulted in substantially more effective energy delivery and retention than the *ad libitum* HAY strategy.

While previous studies have reported improved efficiencies of dietary energy utilization in limit-fed systems for cows (Freetly et al., 2006; Trubenbach et al., 2019; Boardman et al., 2020), application of these findings in group-fed settings may be complicated by intragroup competition for feed (Grant and Albright, 2001), such that less aggressive or subordinate cows consume less than targeted amounts of feed with concomitant overconsumption by others in the group. These behaviors might obviate the reported benefits of these systems, as cows that overconsume are effectively consuming at or near predicted maintenance levels and may not adjust to a different maintenance equilibrium (Freetly and Nienaber, 1998; Freetly et al., 2006) or may not realize improvements in digestion or diet utilization resulting from limited consumption (Galyean et al., 1979; Loerch, 1996). Cows consuming substantially less than the targeted amount may not be able to fully adapt; when Camacho et al. (2014) restricted intake to 60% of predicted maintenance requirements, cows lost weight and BCS throughout the treatment period (i.e., they could not adapt to that degree of limitation). These conditions would be expected to increase intragroup variation in BW and BCS.

In the present study, the within-pen standard deviation of BCS was not materially affected by treatment, but there was some indication that variation in cumulative BW change might increase in pens receiving limit-fed strategies compared to HAY. While these effects were not pronounced, at least not so that they affected overall trends in RE, further evaluation of the application of these strategies in group-fed settings is warranted.

While restricted nutrient intake during gestation has been shown to affect calf birth weight and postnatal growth (Boyd et al., 1987; Long et al. 2010), cows in this study were not fed below maintenance based on observed BW change and estimated RE. As a result, no treatment effects on calf birth weight or growth through d 45 postpartum were observed, suggesting that cow lactation was not affected, and that both the TMR and SEP strategies can sustain production at levels comparable to ad libitum feeding of HAY.

## CONCLUSIONS

Producers lacking the capability to deliver a mixed ration may not be able to capitalize on benefits of an intensified limit-fed system, unless strategies that relieve the requirements of feeding complete diets are available. Delivering limited (i.e., below ad libitum) forage and concentrate separately does not change digestion, and timing of concentrate delivery for deconstructed rations has a little material impact on ruminal fermentation and does not impose the substantial risk of digestive upset using diets of moderate energy density. Limit-feeding a complete or deconstructed diet improved energy retention and sustained cow performance compared to ad libitum hay consumption.

Together these findings indicate that cow-calf producers can choose how an intensified system is implemented to best fit their capabilities so that productivity can be sustained when forage availability is a constraint to more typical grazing systems. These findings extend previous observations about the improvements in the efficiency of maintenance in limit-fed cow-calf systems, and offer attainable strategies for implementation to a wider array of producers.

Variability in cow response may occur as a result of limited feed offerings in group settings; increased variability did not affect mean productivity measurements in this study, but additional research in this area is warranted.

Development of accessible intensified cow systems for operations with different capabilities may improve the long-term sustainability of beef as a global protein source by relieving land constraints and reducing requirements for maintenance during certain periods.
